# Prevalence and impact of preoperative osteoporosis on healthcare utilization and patient-reported outcomes in primary total knee arthroplasty

**DOI:** 10.1007/s00590-025-04418-x

**Published:** 2025-08-04

**Authors:** Alvaro Ibaseta, Ahmed K Emara, Benjamin E Jevnikar, Shujaa T Khan, Ignacio Pasqualini, Oguz Turan, Cleveland Clinic Adult Reconstruction Research, Nicolas S Piuzzi

**Affiliations:** 1 Cleveland Clinic, Cleveland, USA; 2 University of Cincinnati College of Medicine, Cincinnati, USA; 3 Northeast Ohio Medical University, Ravenna, USA

**Keywords:** Primary total knee arthroplasty, Osteoporosis, PROMs, Length of stay, Discharge disposition, Hospital readmission

## Abstract

**Purpose:**

Osteoporosis is a well-recognized risk factor for complications after total knee arthroplasty (TKA). However, the effect of pre-TKA osteoporosis on healthcare utilization and patient-reported outcomes is poorly understood. Here, we characterize the association between pre-TKA osteoporosis and (1) healthcare utilization and patient-reported pain and function outcome measures; and (2) dual X-ray absorptiometry (DEXA) scan* T*-scores and the aforementioned outcomes.

**Methods:**

A prospective cohort of primary elective TKA patients between July 2015 and January 2020 was obtained (*n* = 6318), of which 4922 (77.9%) completed 1-year follow-up. Outcomes included healthcare utilization (prolonged length of stay (LOS) ≥ 3D, discharge disposition (DD), 90-day readmission, and 1-year reoperation) as well as Knee Injury and Osteoarthritis Outcome Score (KOOS) Pain, KOOS-function (PS) and satisfaction.

**Results:**

The prevalence of pre-TKA osteoporosis was 66.8%, of which 28.7% had a DEXA scan and 66.3% were on osteoporosis medications. Medicated osteoporotic patients were independently associated with higher odds of prolonged LOS (Odds Ratio (OR): 1.21 (95% Confidence Interval (CI) 1.02–1.43)) and non-home DD (OR:1.56 (95%CI 1.25–1.95)). Medicated and non-medicated osteoporosis patients were associated with higher odds of 90-day readmission. The odds of failing to achieve MCID or satisfaction were not associated with preoperative OP diagnosis.

**Conclusion:**

Two-thirds of primary TKA recipients had osteoporosis. Among these patients, two-thirds were on medication and one-third had a DEXA scan. Osteoporotic patients are at a higher risk of 90-day hospital readmission, longer hospital stays and non-home discharge. Interestingly, osteoporosis status was not associated with failure to achieve clinically significant improvements or satisfaction at 1 year following TKA.

## Introduction

The burden of osteoporosis, characterized by diminished bone mineral density (BMD) and structural integrity, prominently affects adults over age fifty in the USA. It poses a growing financial concern, with predictions that by 2040, its burden on the US healthcare system could exceed $95 billion [1]. Osteoporosis is widely recognized as a risk factor for complications during and after orthopedic surgery, including periprosthetic fracture and aseptic loosening [2, 3]. Yet, contemporary research fails to adequately address its impact on perioperative healthcare resource utilization and patient-reported outcome measures (PROMs) among total knee arthroplasty (TKA) patients [4, 5]. Additionally, prevalence estimates of osteoporosis among TKA recipients remain variable from 15.5 to 45.5%, with limited characterization of bone quality's influence on surgical outcomes [6, 7]. Furthermore, investigations often neglect the potential impact of concurrent osteoporosis pharmacotherapy on postoperative recovery [3, 8]. Addressing these gaps is crucial, given the increasing prevalence of TKA and its critical role in enhancing patients' quality of life.

The present study sought to address these gaps in the literature by examining a large cohort of TKA patients, clarifying three elements: prevalence of osteoporosis; the correlation between preoperative osteoporosis—both medicated and unmedicated—and healthcare utilization, pain, and function following surgery; and the relationship between bone quality indicators (*T*-scores from dual X-ray absorptiometry (DEXA) scans), perioperative healthcare utilization (length of stay (LOS), discharge disposition (DD), readmission, and reoperation), and PROMs. In doing so, we intend to further characterize the role of osteoporosis in the context of TKA, ultimately guiding improved patient assessment and management protocols.

## Methods

### Study design, setting, and data collection

All osteoarthritic patients who received primary elective TKA between July 2015 and January 2020 using OrthoMiDaS Episode of Care (OME), an institutional prospective data collection system at any of the nine sites within a North American integrated tertiary healthcare system were prospectively screened and subsequently enrolled into an institutional review board (IRB)-approved longitudinal cohort [9–11]. Within the 9 sites, 37 total surgeons were included. OME is a highly accurate and reliable tool for data collection, with capture rates exceeding 97% of all elective orthopedic surgeries conducted within the healthcare system [12–14]. It gathers data on patient demographics, socioeconomic determinants, perioperative and in-hospital metrics, surgical details, as well as both general and joint-specific preoperative and 1-year postoperative PROMs. The current investigation received IRB approval and followed STROBE (strengthening the reporting observational studies and epidemiology) statement guidelines [15].

### Eligibility criteria and population characteristics

All elective primary unilateral TKA within the study period was screened for eligibility. Patients whom underwent non-elective TKA or TKA for oncologic etiology were excluded, as well as those who received contralateral TKA within the 1-year postoperative follow-up interval to avoid potential violation of independence assumptions.

**Fig. 1:**
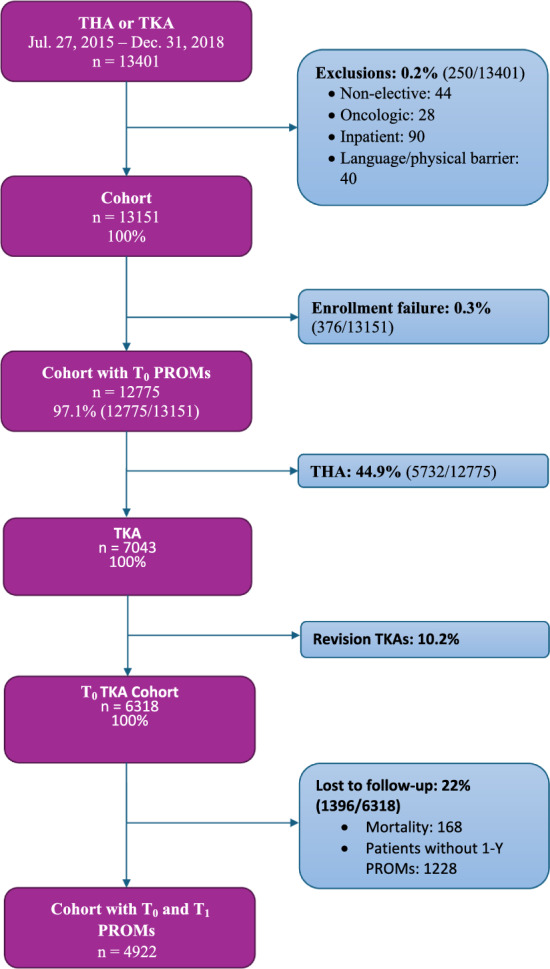
STROBE diagram depicting inclusion criteria of the present patient cohort

Overall, 6318 patients were ultimately included in the analysis (Fig. 1). Among this cohort, 77.9% (*N* = 4922) completed 1-year follow-up. Loss to follow-up at 1 year (22.1%) was attributable to missed visits or incomplete PROMs submission. The median [interquartile range (IQR)] age was 66.0 years [60.0;73.0]. 60.3% were female (*N* = 3810), and 81.8% (*N* = 4928) were white, with a median BMI of 31.8 [27.8; 36.6] (Table 1). 1213 patients had preoperative DEXA scans and were subsequently included in the secondary outcome sub-analyses.

**Table 1:** Patient cohort demographic characteristics, osteoporosis diagnosis, medication status,* T*-score, pain, and function

Age, median [25th; 75th]		*N*
66.0 [60.0;73.0]		6318

Sex (%)		*N*
Male	39.7%	2508
Female	60.3%	3810

Race, (%)		*N*
White	81.8%	4928
Black	15.1%	910
Other	3.04%	183

BMI, Median [25th;75th]		*N*
31.8 [27.8;36.6]		6317

Diagnosis, (%)		*N*
Osteoarthritis	97.20%	6139
Non-osteoarthritis	2.83%	179

Smoking, (%)		*N*
Never	56.0%	3536
Ever	44.0%	2781

Education, median [25th; 75th]		*N*
14.0 [12.0;16.0]		6317

Insurance, *N* (%)		*N*
Commercial/Private/Other	37.80%	1432
Medicaid/Medicare	62.20%	2361

Osteoporosis, (%)		*N*
No	33.2%	2098
Yes	66.8%	4220

Medication, (%)		*N*
No	43.7%	2758
Yes	56.3%	3560

Medicated OP, *N* (%)		*N*
No osteoporosis	33.2%	2098
Medicated osteoporosis	44.3%	2798
Unmedicated osteoporosis	22.5%	1422

Lowest *T*-score, (%)		*N*
< − 2.5	8.66%	105
≥1	39.2%	476
[− 2.5,− 1)	52.1%	632

Lowest *T*-score, Median [25th;75th]		*N*
− 1.30 [− 1.90;− 0.60]		1213

Baseline pain, median [25th;75th]		*N*
38.9 [30.6;50.0]		6318

Baseline PS, median [25th;75th]		*N*
48.8 [38.0;58.0]		6311

OP: Osteoporosis. PS: Function

### Cohort categorization and outcomes

The initial cohort of primary elective TKA patients was stratified by preoperative osteoporosis diagnosis and need for osteoporosis medications (i.e., no osteoporosis, unmedicated osteoporosis, and medicated osteoporosis). Preoperative osteoporosis diagnoses were identified using ICD-9 and ICD-10 codes, which are assigned by physicians or specialized advanced care providers based on World Health Organization (WHO) criteria for osteoporosis diagnosis (*T*-score <  − 2.5; history of low energy hip/spine fracture; osteopenia* T*-scores between − 1.0 and − 2.5 with history of a fragility fracture or a FRAX indicative of 10-year risk of hip fracture > 3% or major osteoporosis fracture > 20%). Patients were classified into the osteoporosis cohort if such a diagnosis existed preoperatively. Similarly, patients were considered on osteoporosis medications if they were prescribed one or more bone mineral density (BMD)-enhancing/preserving medications at the time of surgery.

Primary outcomes included 90-day healthcare utilization (prolonged length of stay [LOS ≥ 3 days], non-home discharge, 90-day readmission and 1-year reoperation) and 1-year PROMs improvement (Knee Disability and Osteoarthritis Outcome Score [KOOS]-Pain, and KOOS-function [PS]). "Treatment failure" for each of the evaluated PROMs was designated as failure to achieve a minimal clinically important difference (MCID) based on validated literature-reported values [16–20]. Patient acceptable symptom state (PASS)—“Satisfaction” was binarily determined based on patients’ responses to the question, “Taking into account all the activity you have during your daily life, your level of pain, and also your activity limitations and participation restrictions, do you consider the current state of your knee satisfactory?”.

Secondary outcomes included the evaluation of the aforementioned metrics in a sub-cohort of TKA patients with available preoperative DEXA scan-based *T*-scores (the difference between patient’s BMD and that of a normal young population divided by the standard deviation of the normal young population [21] and (the number of standard deviations from the mean BMD of a healthy population of the same age, race, and sex [22])*,* which were used as quantitative proxy of bone quality (i.e., BMD). *T*-scores were measured from multiple locations (hip, distal radius, and spine). The lowest *T*-scores were utilized to facilitate statistical analysis.

### Statistical analysis

Median and IQR were calculated for continuous variables including PROMs (KOOS-Pain and KOOS-PS) improvement. Counts and percentages were used to summarize categorical variables including healthcare utilization metrics and achievement of MCID and PASS. Baseline determinants and outcome distribution were characterized using bivariate analyses among patients with and without osteoporosis using Mann–Whitney *U* tests and Chi-square tests for continuous and categorical variables, respectively.

Associations between osteoporosis status and outcomes of interest were evaluated using multivariate regression models, accounting for potential confounders including medication status, age, comorbidity index (CCI), sex, race, BMI, preoperative diagnosis, smoking status, education status and type of insurance. For primary outcomes, the analyzed cohort was categorized based on osteoporosis and medication status (no osteoporosis, osteoporosis with medication, osteoporosis without medication). For secondary outcomes, osteoporosis was continuously evaluated using* T*-scores, with lower scores indicative of poorer BMD. All tests were two-sided with a significance level (alpha) set to 0.05. All statistical analyses were implemented in R version 4.0.3 (R Project for Statistical Computing, Vienna, Austria).

## Results

### Osteoporosis prevalence and severity

An osteoporosis diagnosis was present in 66.8% (*n* = 4220/6318) of patients. Of these, 66.3% (*n* = 2798/4220) were on osteoporosis medication and 28.7% (*n* = 1213/4220) had a preoperative DEXA scan. The median *T*-score was − 1.30 [IQR: − 1.9; − 0.6], with 8.66% having a score of <  − 2.5, 52.1% between − 2.5 to − 1.0 and 39.2% >  − 1.0 (Table 1).

### Postoperative healthcare utilization

LOS ≥ 3 days was observed in 20.0% (*n* = 1064) of patients, 12.0% (*n* = 638) had a non-home DD, 10.2% (*n* = 544) had a 90-day readmission, 1.38% (*n* = 87) had a reoperation, and 0.25% (*n* = 16) had died.

**Fig. 2:**
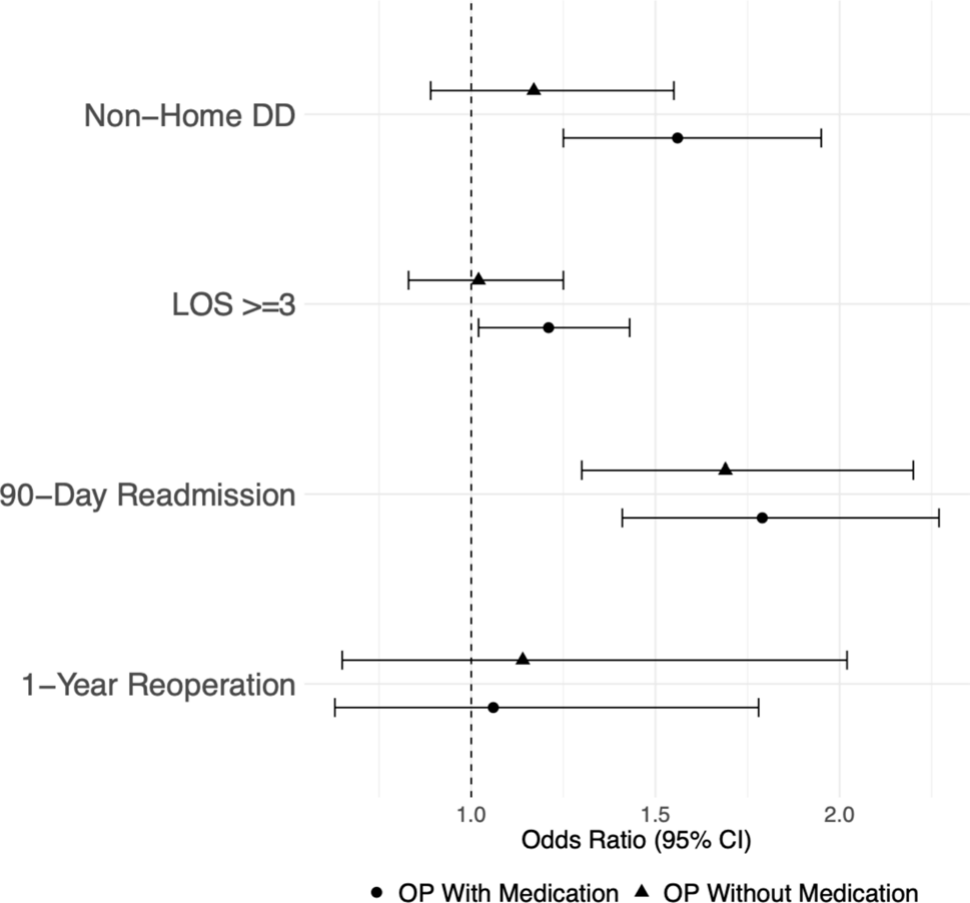
Forest plot depicting healthcare outcomes by osteoporosis medication status. OP: Osteoporosis. DD: Discharge disposition. LOS: Length of stay

A preoperative diagnosis of osteoporosis, medicated or unmedicated, was independently associated with higher odds of 90-day readmission (medicated: OR: 1.79 (95% CI 1.41, 2.27), *p* < 0.001); unmedicated: OR: 1.69 (95% CI 1.3, 2.2), *p* < 0.001). Furthermore, the odds of LOS ≥ 3 and non-home DD were greater among medicated patients (LOS ≥ 3: OP: 1.21 (95%CI 1.02–1.43), (non-home DD: OR:1.56 (95% CI 1.25–1.95). However, unmedicated osteoporotic patients were not associated with LOS ≥ 3 (*p* = 0.88) or non-home discharge (*p* = 0.266). Finally, there was no association between preoperative osteoporosis diagnosis and 1-year reoperation, regardless of medication use (medicated: *p* = 0.838); unmedicated: *p* = 0.643) (Fig. 2).

### 1-Year PROMs

One year following surgery, 93.1% (*n* = 4548) of patients achieved MCID for KOOS-Pain and 84.5% (*n* = 3893) for KOOS-PS. Satisfaction was attained in 85.2% (*n* = 4049) of patients.

**Fig. 3:**
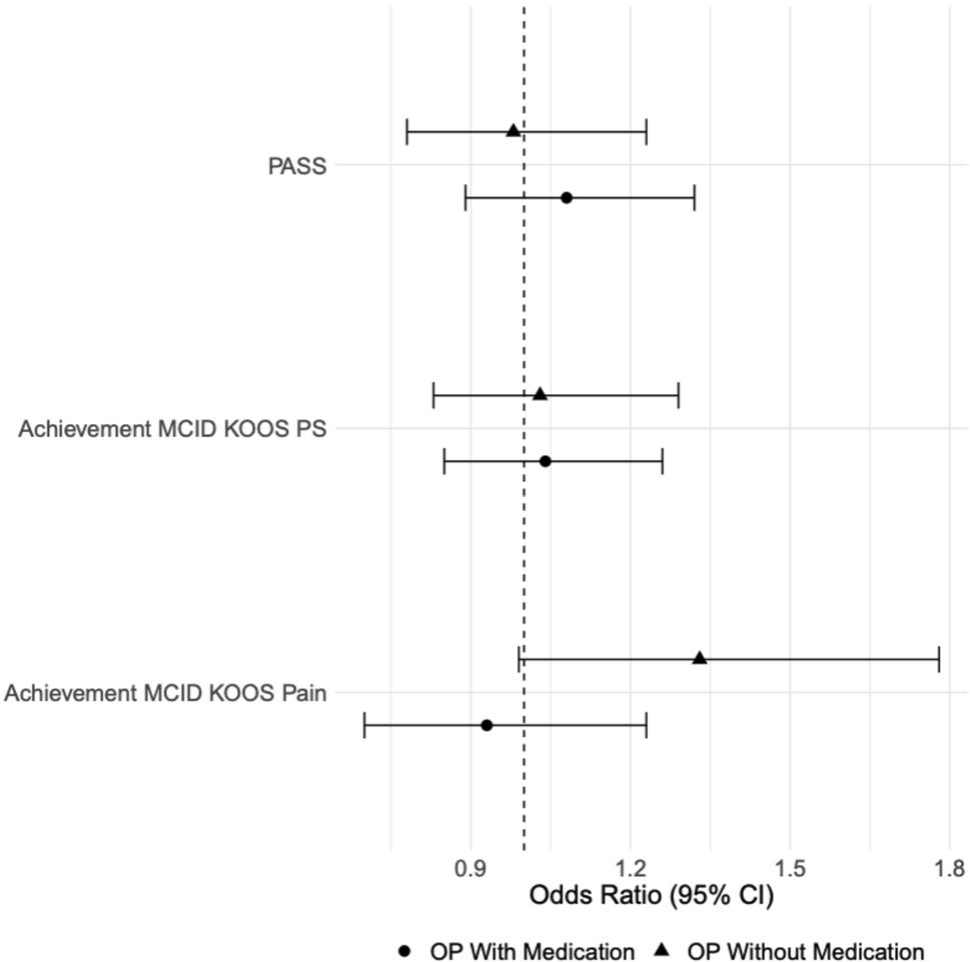
Forest plot depicting 1-year PROMs by osteoporosis medication status. OP: Osteoporosis. MCID: Minimal Clinically Important Difference. KOOS: Knee Disability and Osteoarthritis Outcome Score. PS: Function

After accounting for potential confounders, the odds of failing to achieve MCID for KOOS-pain, KOOS-PS, or satisfaction were not associated with preoperative osteoporosis diagnosis or osteoporosis medication use (Fig. 3).

### Association between 90-day healthcare utilization/1-year PROMs improvement and* T*-scores

**Fig. 4:**
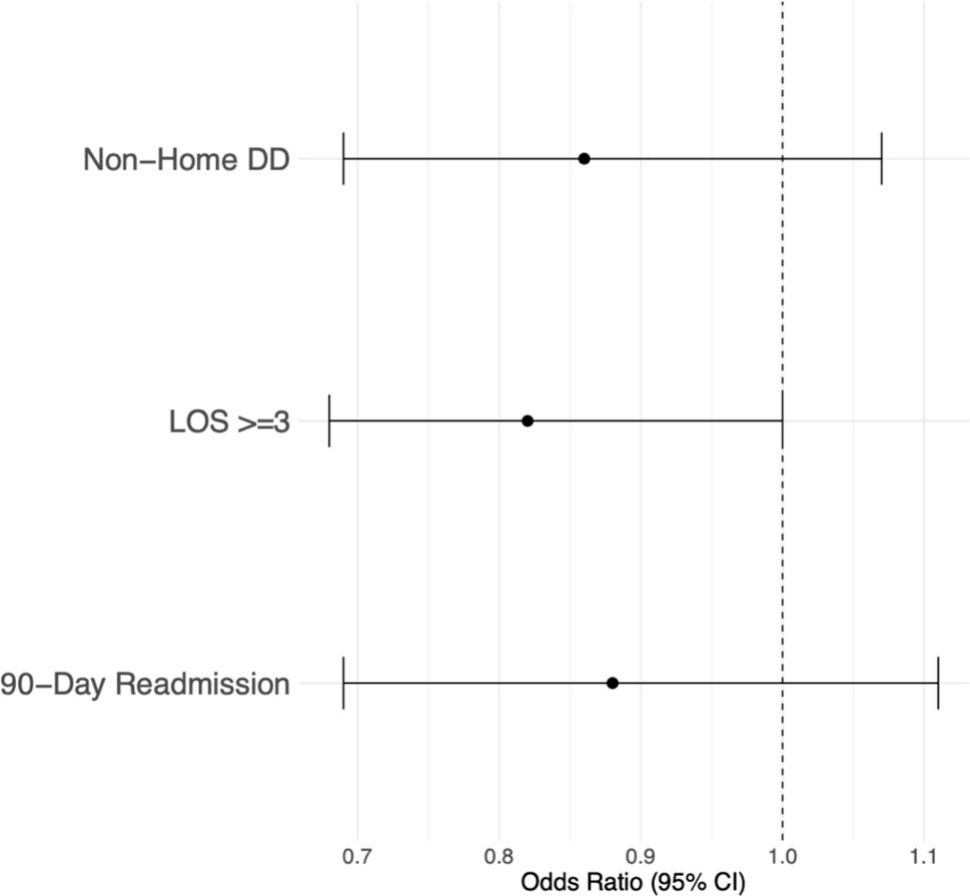
Forest plot depicting healthcare outcomes by lowest *T*-Score. DD: Discharge disposition. LOS: Length of stay

After accounting for potential confounders using multivariable regression analysis, lowest *T*-scores were not significantly associated with odds of prolonged LOS (*p* = 0.052), non-home DD (*p* = 0.184), and 90-day readmission (*p* = 0.281) (Fig. 4).

**Fig. 5:**
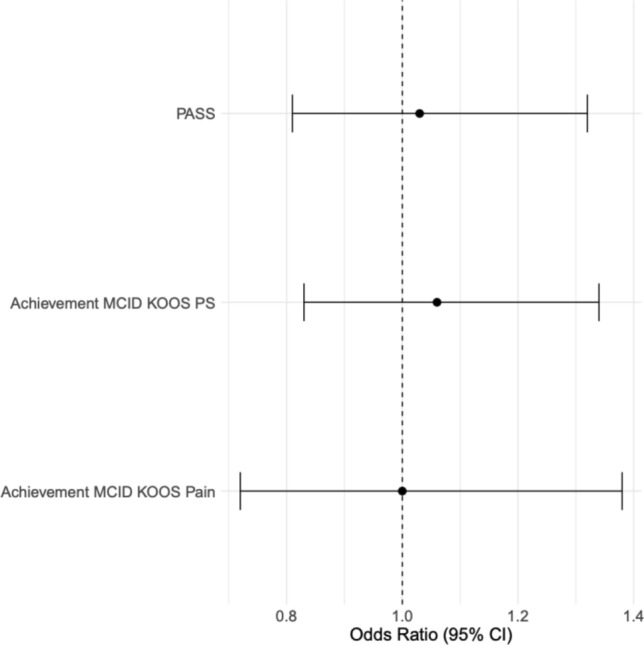
Forest plot depicting 1-year PROMs by lowest *T*-score. MCID: Minimal Clinically Important Difference. KOOS: Knee Disability and Osteoarthritis Outcome Score. PS: Function

Multivariable regression demonstrated no significant association between lowest *T*-scores and MCID achievement of KOOS pain (*p* = 0.987), MCID achievement of KOOS-PS (*p* = 0.650), or satisfaction (*p* = 0.784) (Fig. 5).

## Discussion

In the current value-based healthcare landscape, the high prevalence of osteoporosis among TKA patients poses a significant challenge, as it can lead to increased healthcare costs, prolonged hospital stays, higher readmission rates, and suboptimal patient outcomes. Addressing this issue prior to surgery is crucial for improving the quality of care and reducing the financial burden on healthcare systems. Here, 66.8% of TKA patients had osteoporosis, yet a considerable proportion of these patients were not prescribed osteoporotic medications, and less than one-third had preoperative DEXA scanning. Osteoporotic patients were at 70% higher risk of 90-day hospital readmission, and medication-requiring osteoporosis patients were at a 20 and 50% greater risk of prolonged LOS and non-home DD, respectively. Osteoporosis was not associated with clinically significant improvements in pain, function, or satisfaction at 1 year.

The prevalence of osteoporosis among this patient cohort differs slightly from traditionally recognized rates of 15.5–45.5% in TKA recipients [6, 7]. This discrepancy may be reflective of diagnostic and coding practices specific to the study’s healthcare system or geographical variability, as well as the fact that this study was conducted at a tertiary institution that more frequently treats patients managing multiple comorbidities. Additionally, our institution practices thorough preoperative evaluation and treatment of osteoporosis leading up to surgery, which may have promoted otherwise undetected osteoporosis prevalence, thereby accounting for these results. Despite the higher prevalence of preoperative osteoporosis diagnosis, a considerable proportion of these patients were not prescribed osteoporotic medications, and less than one-third had preoperative DEXA scanning. Consistent with current literature, Wang et al. [23] analyzed 505 total joint arthroplasty (TJA) patients at a single academic institution, finding that 90% of TJA patients did not receive any form of pharmacological treatment for osteoporosis. Similarly, Xiao et al. [7] reported less than a third of TJA patients with osteoporosis received treatment. Regardless of the underlying cause, this high prevalence accompanied by low diagnostic testing and medicated management emphasizes the clear need for heightened awareness and vigilance concerning osteoporosis in pre-TKA patients.

Both medicated and unmedicated osteoporotic patients were at greater risk of 90-day readmission, suggesting that osteoporosis, regardless of treatment status, presents a significant risk factor for postoperative complications or the exacerbation of comorbid conditions leading to readmission. The isolation of this trend in post-TKA LOS ≥ 3 and non-home DD among medicated osteoporotic patients suggests two possible interpretations. Firstly, the need for osteoporosis medication may indicate more severe baseline bone quality, which could predispose patients to complications in the perioperative and postoperative phases of surgical treatment. McDonald et al. [24] investigated the association between the number and type of pre-operative medications and postoperative complications among geriatric patients undergoing hip fracture surgery, establishing a strong and linear relationship between the number of medications taken prior to surgery and postoperative complications. While their findings did not isolate the relationship between postoperative complications and osteoporosis medication specifically, our findings do warrant further study. Secondly, these findings may reflect suboptimal therapeutic management or adherence to osteoporosis treatments—a frequently cited challenge in the current literature. Deng et al. [25] investigated predictors for discontinuation of osteoporosis medication, finding over half of patients self-discontinuing during intended treatment. Additionally, while it is possible that older age and medical comorbidities may influence LOS, our multivariable models adjusted for both factors, reducing the likelihood that these variables might explain the observed association. Instead, we suspect that hospital-level factors may have contributed to these findings, including institutional discharge protocols or rehabilitation resource availability. These possibilities underscore the necessity for rigorous preoperative assessment and optimization of osteoporosis to help better manage or mitigate these risks.

Osteoporosis status and lowest *T*-scores were not associated with failure to achieve clinically significant improvements, as measured by MCID, in pain, function, or satisfaction at 1 year. This suggests that osteoporotic patients can achieve similar clinically meaningful benefits from TKA as those without osteoporosis. Our findings are consistent with those of Meyer et al. [8], who reported comparable postoperative PROMs among 1306 osteoporotic and non-osteoporotic TKA patients. The collective findings from our study and that of Meyer et al. suggest that while osteoporotic patients may have worse preoperative health-related quality of life, they can still achieve significant and clinically meaningful improvements in both joint-specific and generic health status outcomes following TKA. While these results support the fact that patients with osteoporosis can expect clinically significant improvements from TKA, it is still crucial for clinicians to carefully assess and optimize bone health in osteoporotic patients prior to surgery and implement strategies to mitigate the increased risk of adverse events associated with osteoporosis in the perioperative setting.

This study is not without limitations. 22.1% (*N* = 1396) of patients were lost to 1-year follow-up, and only 29% (*N* = 1213) of osteoporotic patients had preoperative DEXA scanning and consequently could be included in secondary outcome analysis. With only a third of patients having underwent a preoperative DEXA scan, stratification of patients by osteoporosis severity was limited among patients included. Loss to follow-up may introduce attrition bias; however, the large overall sample size and high capture rate (78%) mitigate this limitation. The observed 22% of patients lost to 1-year follow-up is likely multifactorial, reflecting both patients who may have experiences satisfactory outcomes and felt no need to engage in further follow-up, as well as those who may have been less satisfied and chose to not complete postoperative surveys. Importantly, this follow-up rate is consistent with previously reported rates in similar large-scale registry studies [26]. The low number of patients with DEXA scans could also be because some patients had their scans done outside our healthcare system, which we were unable to capture. While the proportion of patients who had a preoperative DEXA scan is notably low compared to current guidelines, this patient cohort is among the largest to date in evaluating the impacts of osteoporosis on TKA outcomes, and therefore this limitation is highly unlikely to influence our findings. Another limitation is the lack of data on time since osteoporosis diagnosis. Since these data were not recorded, duration-related effects on outcomes could not be assessed. Temporal categorization of osteoporosis diagnosis can be potentially beneficial in future studies. The authors were unable to account for osteoporosis medication types, duration of usage, or compliance, a limitation frequently reported among studies that categorically evaluate medication use. Based on our findings, it may be useful to consider quantifying osteoporosis medication compliance when assessing TKA outcomes in future studies. Additionally, this cohort was obtained from a tertiary health-care system which may inherently introduce selection bias to patients of greater complexity. Another limitation is the lack of longer-term follow-up beyond 1 year. However, prior studies have demonstrated that PROMs tend to stabilize after the first postoperative year, suggesting that longer-term follow-up may offer limited additional insight [27, 28]. Furthermore, data regarding implant manufacturer and fixation technique were not captured and likely varied based on surgeon preference and institutional protocol. Another limitation to consider is the potential for response bias. Although validated, PROMs may be influenced by reporting and recall biases, which could impact the accuracy of subjective outcomes. Furthermore, the use of a single-item PASS measure may be subject to response bias, as patients may feel inclined to respond positively. However, it is worth noting that the PASS is a highly recognized and validated questionnaire, as supported by ample literature [20, 29, 30]. Lastly, although these diagnoses were placed by providers trained based on WHO criteria of diagnosis, osteoporosis was determined using preexisting chart-coded diagnoses which presents risk of human and code errors.

Future research should focus on developing clear guidelines for osteoporosis management in the perioperative TKA setting, evaluate the cost-effectiveness and patient-centered outcomes of interventions, and establish a consensus on the timeline and strategy for postoperative osteoporosis care continuity. Future studies investigating whether the associations observed at 1 year persist over longer-term follow-up intervals would also be valuable. Additionally, randomized controlled trials investigating the efficacy of bone-modifying agents in the perioperative period could offer evidence-based approaches to improving outcomes for these patients. Finally, exploration of genetic factors and biomarkers of bone metabolism may also provide further insights into individual patient risk stratification and customized therapeutic strategies.

## Conclusion

Approximately two-thirds of primary TKA patients had osteoporosis. However, only two-thirds of these patients were on osteoporosis medication, and less than a third had a DEXA scan. Osteoporotic patients are at a 70% higher risk of 90-day readmission, and medication-requiring osteoporosis patients are at a 20 and 50% greater risk of prolonged LOS and non-home DD, respectively. There was no association between the achievement of clinically significant improvements and osteoporosis status. These findings suggest that while osteoporosis is a marker of increased perioperative risk, it should not deter patients or surgeons from pursuing TKA. Proactive screening and optimization of bone health may help mitigate early risks without compromising outcomes at 1 year.

## Data Availability

No datasets were generated or analysed during the current study.
